# Performing is adapting: an ecological dynamics framework for skill learning *in* development in handball

**DOI:** 10.3389/fspor.2026.1869713

**Published:** 2026-08-07

**Authors:** Christian Thue Bjørndal, Mark O’Sullivan

**Affiliations:** Department of Sport and Social Sciences, Norwegian School of Sport Sciences, Oslo, Norway

**Keywords:** affordances, ecological dynamics, handball, representative learning design, skill adaptation

## Abstract

This conceptual paper proposes an ecological dynamics perspective as a theoretically coherent and practical framework for handball coaching, education and sport science. Handball is a fast-paced, complex sport in which skilled performance cannot be reduced to the execution of stored movement programmes. Drawing on Bernstein's analysis of the degrees of freedom problem and the principle of repetition without repetition, Newell's model of interacting constraints, and Gibson's ecological theory of direct perception and affordances, we articulate a conceptualisation of skill adaptation in which the player is understood as a complex, self-organising system continuously coupled to their environment. Skill in handball, on this account, is not a fixed property of the player but a relational one, realised in the performer-environment system through the coupling of the player's perceptual-motor capacities and the affordance structure of the performance context. A central intention of this paper is to dissolve the traditional analytical distinction between technical, tactical, physical and psychological components of skilled performance. In the living, adapting player, these are not separable dimensions but mutually constitutive features of a single, integrated perceptual-motor system. Functional skill is an emergent phenomenon of what conventional sport science has artificially decomposed into discrete components. The paper makes two interrelated contributions: a conceptual account of skill adaptation for handball across individual, dyadic, and collective team levels; and a methodological account proposing a task design model grounded in representative learning design, repetition without repetition, and constraints manipulation, applicable across the full developmental arc from children's first encounters with handball to elite performers.

## Prologue

As a young handball coach, I (the first author) completed my master's degree in sport science and coaching from the Norwegian School of Sport Sciences in 2009. I was already coaching professionally at the second tier of Norwegian handball and pursuing my European Handball Federation professional coaching licence. My education had given me considerable knowledge across the scientific disciplines considered relevant to handball—physiology, biomechanics, psychology, and training methodology—yet I found myself without a coherent theoretical understanding of skill learning in the sport. The curriculum had emphasised isolated training modalities and the physiological adaptations associated with them, with little attention to the quality of handball performance as an integrated whole.

In practice, I could often distinguish effective session design from what was not—certain exercises felt right, others felt hollow—but I lacked the conceptual clarity to articulate why. When I became a university lecturer some years later, I turned to the literature on motor learning and control as it had been applied in sport settings. It was here that I encountered the first edition of *Dynamics of Skill Acquisition* (1). My introduction to what is now widely referred to as an ecological dynamics conceptualisation of skill adaptation and learning proved formative. For the first time, I had a theoretical language capable of integrating the fragmented disciplinary knowledge I had accumulated—one that could account for the dynamics of skill acquisition in handball as a coherent, meaningful whole, applicable from children encountering handball for the first time to professional players refining their craft. This paper is a result of that intellectual development.

## Introduction

The purpose of handball is to outscore your opponent. Skill adaptation is therefore about learning and refining the movement and problem-solving solutions that, individually and collectively, help players score or prevent goals. Yet the game makes extraordinary demands on those solutions. Handball is a fast-paced, full-contact sport characterised by continuous transitions between attack and defence, rapid decision-making under physical and temporal pressure, and the perpetual co-adaptation of individual players and collective units in response to an ever-changing opposition (2–4). A skilled back court player driving towards a 6:0 defensive block does not execute a pre-planned movement sequence: they perceive, in real time, the positions and momentum of defenders, the movement of teammates into space, and the emerging geometry of the situation, and they act accordingly. A skilled pivot player receiving the ball with their back to goal under contact from a defender draws simultaneously on haptic, visual, and vestibular information to compose a movement solution that may bear little resemblance to what emerged in the previous possession. Skill in handball is not the stable reproduction of fixed techniques. It is the continuous, context-sensitive reorganisation of perception and action in response to shifting constraints. Performing is adapting.

Yet the dominant frameworks through which skill development has traditionally been understood in sport science, and by extension in handball coaching education, have struggled to account for precisely this quality (5). Indeed, these approaches persist due to entrenched beliefs and coaches' attachment to methods they “succeeded” with, raising the question of how to support practitioners in updating outdated ideologies (6, 7). From the behaviourist stimulus-response models of the early twentieth century, through Fitts' three-stage model of motor learning and Schmidt's schema theory, the prevailing view has been that skill acquisition is fundamentally a process of storing and increasingly refining motor programmes in the brain, to be retrieved and executed in response to environmental stimuli (8). The brain is conceived as a computational device, and skill development is measured by the progressive reduction of variability as the learner moves from a cognitive to an autonomous stage of performance. Skill is, in this sense, something a player has, stored and available for deployment, rather than something that emerges from the dynamic interaction between the player, the task, and the environment^1^.

The practical consequences of this orientation have been significant and, we argue, limiting. If skill is a stored programme, then training logically becomes a matter of repeatedly activating and refining that programme in isolation from the full complexity of the game. This has encouraged a reductionist approach in which physical, technical, and tactical capacities are treated as separable components to be developed independently, an approach in which agility ladder drills, isolated throwing repetitions, and set-piece tactical rehearsal each address a discrete dimension of performance. It struggles to explain why movement solutions in handball are invariably variable; why effective players continuously adapt their technique within and across match situations; and why skills trained without the perceptual information of the performance context so often fail to transfer when it matters most (10, 11).

This paper proposes that an ecological dynamics framework offers a theoretically coherent and practically productive alternative. Drawing on Bernstein's work on the degrees of freedom problem and the principle of repetition without repetition, Newell's model of interacting constraints, and Gibson's ecological theory of direct perception and affordances, we articulate a conceptualisation of skill adaptation in handball that treats the player not as a programme-executing machine, but as a complex, self-organising system continuously coupled to their environment. Skill adaptation, in this framework, is understood as the progressive attunement of perception-action couplings to the informational constraints of the game, a process operating simultaneously at the level of the individual player, the attacker-defender dyad, and the collective team system (12). The paper makes two interrelated contributions: a conceptual account of skill adaptation across individual and team levels, illustrated with handball examples throughout; and a methodological account, proposing a user-friendly task design model grounded in the theoretical framework and applicable across the full developmental arc, from children's first encounters with the sport to elite performers refining their craft.

## Theoretical foundations: ecological dynamics

Ecological dynamics is not a single theory but a theoretical framework drawing together insights from dynamical systems theory, ecological psychology, and the biology of movement (13). Its foundational claim is that skilled movement cannot be explained by reference to the brain alone, stored representations or hierarchical executive control, but must be understood as arising from the continuous, dynamic interaction between a performer, the task, and the environment. Three intellectual contributions are foundational to the framework as applied in this paper: Bernstein's analysis of the degrees of freedom problem, Newell's model of interacting constraints, and Gibson's ecological theory of direct perception and affordances.

### Bernstein and the degrees of freedom problem

Nikolai A. Bernstein's (1896–1966) central insight was that the redundancy of the human movement system is not a problem to be suppressed but a resource to be exploited. The body possesses an almost limitless number of anatomical degrees of freedom—the many joints and muscle groups that can contribute to any given movement—and any trajectory through space can be achieved through an enormous variety of combinations of joint angles, velocities, and accelerations (14). For any motor task, there is not one correct solution but a multiple number of movement configurations that could, in principle, achieve the same functional outcome. Bernstein's question was: what determines which configuration the movement system adopts in each moment?.

His answer, developed through meticulous kinematic analyses of skilled workers performing repetitive industrial tasks, most famously blacksmiths striking a chisel, was that the movement system resolves this problem through *self-organisation*: the spontaneous emergence of coordinated, functional movement patterns from the interaction of the organism's many subsystems with the demands of the task and the properties of the environment (15). What guides this self-organisation is not a blueprint specifying what each joint should do, but the functional goal itself, the outcome the system is organised around achieving.

This insight led directly to what Bernstein captured in his celebrated formulation of *repetition without repetition*. The blacksmith striking the same chisel repeatedly does not reproduce identical joint kinematics on successive blows; what is reproduced is the functional outcome—accurate, forceful contact—through continuously varying movement solutions (16). Variability across repetitions is not imprecision or noise; it is the signature of a movement system that is self-organising around a goal rather than executing a fixed sequence (17). Far from being noise to be minimised, variability is the very medium through which the system remains responsive to the fact that task conditions—the precise angle of the chisel, the fatigue state of the muscles, the micro-variations in grip and stance—are never quite the same twice. The practiced performer doesn't repeat a fixed movement; they repeatedly engage in the process of solving the movement problem, finding a functional solution each time through slightly different coordination.

The implications for handball are immediate and far-reaching. Biomechanical analyses of the handball jump shot and different throwing techniques consistently reveal that even highly skilled back court players produce substantial kinematic variability across repeated attempts—in run-up speed, take-off angle, trunk rotation, and arm trajectory—while achieving consistent functional outcomes in ball speed and accuracy (18–20). This is not technical inconsistency; it is the self-organising movement system responding appropriately to the fact that no two shooting situations present the same configuration of constraints (21). The defensive positioning, the angle of approach, the player's momentum and fatigue state, the goalkeeper's starting position, all shift continuously across attempts, and the movement system shifts with them, composing a new coordinative solution each time from the interacting demands of that specific situation. The hallmark of expertise in handball, as Bernstein's framework predicts, is therefore not the reproduction of a fixed and polished technique, but the capacity to find a functional movement solution across a wide range of situational variations—what Vereijken et al. (22) would later characterise as the progressive freeing of degrees of freedom that defines advanced skill.

### Newell's model of interacting constraints

If Bernstein identified the problem and proposed self-organisation as the answer, Karl Newell's model of interacting constraints (23) provided the theoretical framework for understanding what self-organisation is organising *in response to*. Newell proposed that movement emerges from the interaction of three categories of constraint: those residing in the *organism* (the individual performer), those inherent to the *task*, and those presented by the *environment*. No single category is sufficient to explain a movement solution; it is the dynamic interplay among all three that channels the movement system towards coordinative patterns (24).

Organismic constraints include morphological characteristics: height, body mass, limb lengths, muscle architecture; as well as functional properties such as strength, coordination, perceptual acuity, fatigue state, and emotional disposition. Task constraints are the goals, rules, and implements that define what a performer is trying to achieve and how: the three-step rule, the six-metre zone, the dimensions and weight of the ball, the rules governing body contact. Environmental constraints are the physical and sociocultural properties of the performance setting: the surface underfoot, crowd noise, and the broader cultural norms, beliefs and expectations that shape how the game is played in each context.

Critically, constraints do not act in isolation. A change in any one constraint can alter the movement solutions that emerge from the system as a whole. This is what gives Newell's model its practical power: it frames the task of the coach not as the instruction of correct technique but as the *design of constraint configurations* that invite, channel, and support functional movement solutions appropriate to the performance context (25). Constraints, in this sense, are the coach's primary session design tool. In essence, all coaching is constraints based, as coaches and players operate within a system of constraints that shape learning and performance. The key difference lies in *how* those constraints are manipulated (26).

**Figure 1:**
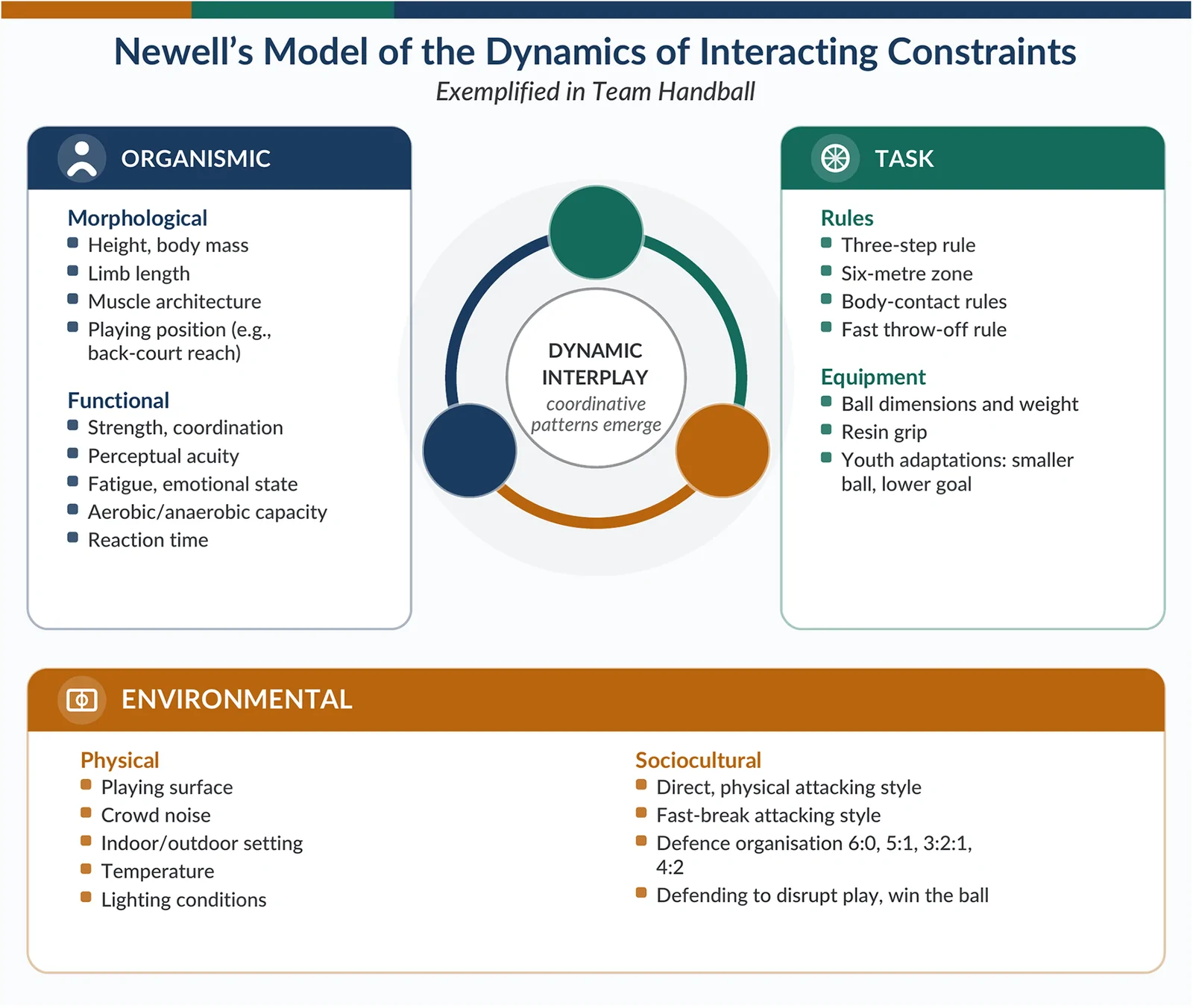
Newell's model of the dynamics of interacting constraints, exemplified in team handball. According to Newell (23), behaviour, including movement solutions and decisions in handball, emerges from the dynamic interaction of three categories of constraint: *organismic constraints*, comprising the morphological (e.g., height, body mass, limb length, muscle architecture, playing position) and functional (e.g., strength, coordination, perceptual acuity, fatigue and emotional state, aerobic/anaerobic capacity, reaction time) characteristics of the performer; *task constraints*, encompassing the rules (e.g., the three-step rule, the six-metre zone, body-contact rules, the fast throw-off rule) and equipment (e.g., ball dimensions and weight, resin grip, youth adaptations) that define the performance challenge; and *environmental constraints*, encompassing the physical (e.g., playing surface, crowd noise, indoor/outdoor setting, temperature, lighting) and sociocultural (e.g., attacking style, defensive organisation) properties of the performance setting. The continuous, dynamic interplay of these three constraint categories channels the movement system toward coordinative patterns.

Handball offers rich examples of constraint interactions across all three categories (see Figure 1). At the individual level, a tall back court player with long arms operates under different organismic constraints than a shorter, faster player of equivalent skill; both will find different movement solutions to the same shooting situation. As fatigue accumulates across a match, organismic constraints shift—muscle force production declines, attentional resources narrow—and movement solutions shift accordingly: the player who generates a powerful standing throw in the first half may rely more on body rotation and momentum transfer in the final minutes. At the task level, the rules of handball are not merely regulatory—they are constitutive of the perceptual landscape players navigate. The three-step rule shapes the rhythm of attacking play and the timing of defensive pressure. The six-metre zone funnels shooting opportunities to specific areas and distances. The rule permitting body contact within defined limits opens a range of movement opportunities for both attackers and defenders: the pivot's body-screening actions, the defender's use of chest and arms to channel an attacker's path, that would not exist in a non-contact sport. Equipment constitutes a further layer of task constraint whose effects are visible at the level of the game itself. The progressive development of resin-enhanced balls with superior grip has expanded the range of throwing techniques available to skilled players, enabling wrist-snap releases, spin shots, and fast-throws executed with minimal wind-up from distances and angles that would previously have required a full throwing action. Performance analysis research has documented corresponding shifts in game play and team performance, e.g., the full implementation of the fast throw-off in 2001 led to a significant increase in attack tempo and fast-break frequency (3, 27). Modifying these task constraints in training changes the movement solutions players explore and the affordances they learn to perceive, which is precisely why small-sided and modified games are such powerful vehicles for skill development when designed with specific intentions in mind (28).

Environmental constraints extend beyond the physical properties of the court. The sociocultural environment, e.g., the norms, expectations, and tactical traditions of the handball culture in which a player develops, constitutes a powerful and often underappreciated constraint on movement and action (29, 30). Players who grow up in handball cultures prioritising direct, physical play through the centre of the defence will develop different perceptual-motor habits than those raised in cultures emphasising wide play, fast breaks, and positional fluidity.

### Gibson's affordances and perception-action coupling

The third foundational pillar of the ecological dynamics framework is the ecological psychology of James J. Gibson (1904–1979), whose concept of affordances and theory of direct perception provide the account of how performers relate to their environments that Bernstein's and Newell's frameworks implicitly require. Gibson's central claim was that perception is *direct*. The environment contains information—structured patterns of light, sound, pressure, and flow that specifies its properties for an active, moving organism without the need to construct internal representations, retrieve stored knowledge, or engage in inferential processing (31). The perceiver does not build a model of the world and then act on the model; they perceive the world directly, in terms of what it offers or invites for action, and act accordingly.

Central to Gibson's framework, and to the ecological dynamics approach, is the principle of perception-action coupling: the continuous, mutually constitutive relationship between what a performer perceives and what they do. Rather than treating perception and action as sequential stages—perceive first, then respond—Gibson understood them as a continuous loop: perception guides action, and action generates the perceptual information that shapes further perception (32). This was highlighted by Gibson (31) when he stated, “we must perceive in order to move, but we must also move in order to perceive.” It follows that perception cannot be developed independently of action, and action cannot be trained without the perceptual information that shapes it in performance, a point with fundamental consequences for how learning environments must be designed, which we develop in the methodological section below.

A key concept here is the notion of *affordances*: the action possibilities that the environment offers to a particular organism with particular properties and capabilities (33). Affordances are relational properties, emerging at the interface between organism and environment (34). A staircase affords climbing for an adult but not for an infant; a passing lane affords penetration for an attacker with sufficient speed but not for one who is slower or less well-positioned. The same physical configuration presents different affordances to different performers, and different affordances to the same performer at different moments—fresh vs. fatigued, positioned centrally vs. out wide, taller vs. shorter player, skilled shooter vs. skilled 1v1 player. In handball, affordances illuminate tactical and technical behaviour in ways that the language of motor programme selection cannot capture. The gap between two defenders is not simply a spatial interval that an attacker detects and then decides to exploit: it is an affordance for penetration that emerges dynamically from the relative positions, velocities, and body orientations of all the players involved, and that exists only for an attacker with the speed, body angle, and ball-handling capacity to use it. A skilled back court player does not see a gap and then decide to drive; they perceive the gap *as* an opportunity to drive, and movement is already underway before anything that could be called a “decision” has been made. The goalkeeper reading a shooter's approach exemplifies the same principle. Research has shown that expert goalkeepers rely heavily on kinematic information from the shooter's run-up, trunk orientation, and arm preparation—information available well before ball release—to initiate their save response (35, 36). The goalkeeper who attunes to earlier and more subtle sources of information in the shooter's kinematics gains crucial milliseconds. This attunement is itself a skill, developed through extensive experience of perceiving and acting in representative environments.

It is important to note, however, that affordances in team sport are not merely physical or body-scaled properties fixed in the environment. They are entangled in a particular way of life (normative practices, values) illustrating a particular social, the shared agreement in reactions that makes rule following and gesture possible, which in turn is inflected by particular social, cultural and historical conditions (26). More directly, they are constituted within the sociocultural practices of the game, the tactical traditions, positional conventions, and rule structures that collectively shape what players come to perceive as opportunities to act (37, 38). The aforementioned fast throw off in handball, for instance, did not become an affordance for attacking play simply because the rules permitted it after 2001; it became one as tactical cultures developed, as coaches designed practices around it, and as players accumulated experience of reading and exploiting the momentary disorganisation of a recovering defence. What counts as a perceivable and actionable opportunity in handball is always, in part, a product of the game's sociohistorical development—which is precisely why players who are deeply embedded in a particular handball culture perceive affordances that newcomers to the game, however physically capable, do not yet see see also (39). So, the affordance isn't just about what is physically possible; it's about what is *meaningful* and *appropriate* within that particular way of playing the game (40).

## Individual skill and team-level adaptation

Individuals learn to adapt their movements to the performance context through a process that Ingold (41) describes as *enskillment*: the development of perceptual and motor capacities not through the transmission of explicit knowledge, but through sustained, attentive engagement with a skilled environment^2^. Enskillment occurs through *dwelling*: immersive, practice-embedded participation in which the learner is not instructed in a technique from the outside but grows into a way of perceiving and acting from the inside (42–44). In an ecological dynamics interpretation of handball, the skilled player does not acquire a repertoire of stored techniques; rather, they become progressively attuned, through thousands of hours of contextually embedded practice, to the specific informational textures of the game.

This process maps directly onto Bernstein's concept of *dexterity*: defined not as the execution of technically correct movements but as the ability to find a motor solution for any situation: the capacity to cope with an emerging motor problem in conditions where fixed, pre-established templates are inadequate (16). Dexterity is what separates the player who can execute a reliable jump shot in isolation from the player who can generate a goal-scoring movement solution from an irregular approach angle, under physical pressure, with a goalkeeper who has already read the first option. It is not a single skill but a meta-capacity: the flexible, situation-responsive organisation of the perceptual-motor system.

Because functional adaptations occur across multiple timescales, different capacities develop at different rates. Acute states such as motivation, arousal, and fatigue develop within a session or match, and can meaningfully enhance or inhibit both learning and performance (45–47). Capacities such as strength, endurance, and coordination emerge over weeks or months of targeted exposure (4, 22, 48). Anthropometric characteristics and higher-order perceptual attunements, the capacity to read complex game patterns, develop across many years of enskilled practice (35, 49, 50). Crucially, these adaptations cascade (51). When a young player acquires the physical strength to generate sufficient ball speed without compromising movement control, new shooting options become available; when they develop the coordination to execute a jump shot from both feet or the weaker side, the defender's task becomes fundamentally more complex, opening further affordances. Adaptation is autocatalytic: each functional advance enlarges the affordance landscape, and the newly available affordances invite further exploration and further learning (52).

Physical capacities in handball are not merely fitness components; they are organism constraints that directly limit the range of movement solutions available (53, 54). A pivot player lacking sufficient trunk rotation will find that certain turning-and-shooting solutions simply do not appear as available affordances; the movement system cannot compose what the body cannot execute. Equally, limited perceptual attunement renders technical skills functionally useless in match conditions. A player who can produce a technically polished step-shot in isolation but cannot read the timing of a defensive block cannot deploy that technique effectively under competition conditions. These observations dissolve the traditional analytical distinction between technical, tactical, and physical components of skill: in the living, adapting player, they are not separable dimensions of performance but mutually constitutive features of a single, integrated perceptual-motor system (21). Functional adaptation, i.e., skill, in its fullest sense, is therefore an emergent phenomenon of what conventional sport science has decomposed into individual components (55).

In team sport, this logic of emergence extends from the individual to the collective. Players do not merely adapt to static environmental configurations; they co-adapt continuously to each other (56, 57). Research on dyadic and collective coordination in team sports has demonstrated that attacker-defender dyads exhibit the properties of self-organising dynamical systems (58): stable coordination modes emerge and dissolve as the relative positions, velocities, and orientations of the two players shift, and transitions between modes, e.g., the moment a defender commits and an attacker drives past, correspond to bifurcation points in the dynamical landscape of the encounter (59–61). At the team level, collective coordination in handball, i.e., the synchronised movement of a defensive line, the timing of a second-wave attack, the spatial organisation of a fast break, emerges from the local interactions of individual players responding to shared perceptual information, rather than from the execution of a centrally prescribed tactical plan (62–64).

## Methodological implications

**Figure 2:**
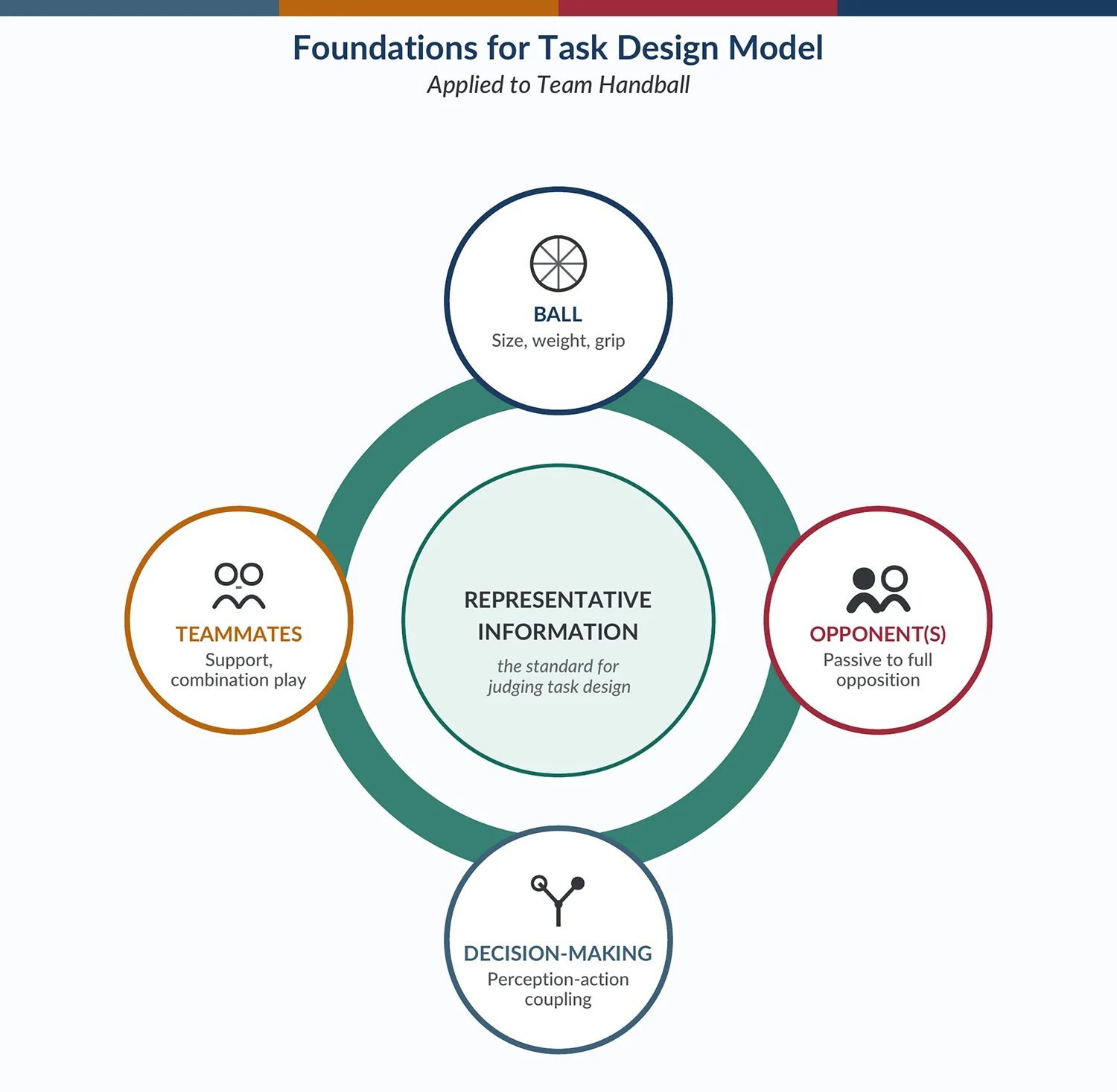
The Foundations for Task Design model applied to team handball, adapted from O'Sullivan et al. (26). The model organises four interdependent parameters: ball; opponent(s); teammates; and decision-making, understood here as perception-action coupling — around a central criterion of *representative information*: the requirement that practice tasks generate the perceptual information that players must attend to and act upon in competitive handball. Each parameter offers distinct affordance-shaping possibilities for the coach; modifications to any one parameter propagate through the others, reshaping the informational and movement demands of the task as a whole.

The theoretical principles outlined above carry direct and fundamental implications for how practice and training environments in handball should be designed. In what follows, we highlight three principles: (a) representative learning design; (b) repetition without repetition; and (c) constraints manipulation. These are not independent prescriptions but mutually constitutive design principles, each shape and regulates the others. Together, they find practical expression in our adapted version of the Foundations for Task Design model (see Figure 2), a handball-specific operationalisation of constraints-based task design that identifies four coupled parameters, i.e., ball, opponent(s), teammates, and decision-making, against the overarching criterion of representative information (65). The model is introduced here not as a replacement for the theoretical principles but as a pedagogical language through which coaches can plan, evaluate, and adapt practice tasks considering those principles.

### Representative learning design

If skill is constituted by the coupling of perception and action within a specific affordance landscape, then the quality of a learning environment must be judged not by whether it superficially resembles the performance context, but by whether it preserves the specific information-movement couplings players will encounter in competitive match play. This is the core insight of what Pinder et al. (66) termed representative learning design, a concept rooted in Egon Brunswik's (67) original notion of representative experimental design. Critically, Pinder et al. (66) distinguished representative design from the weaker concept of ecological validity: functional equivalence, not fidelity, is the criterion. What matters is the degree to which practice tasks maintain the perception-action couplings and affordance structures constitutive of skill itself.

Perceptual information is therefore not incidental to practice design; it is the substrate of skill. In handball, specificity cannot be reduced to technical resemblance. A shooting drill against a passive or stationary defender may look like handball, but it does not informationally behave like handball. The defender's movement kinematics, the spatial pressure of an active opponent, postural cues signalling when a gap is opening or closing, and timing constraints imposed by a goalkeeper reading the same situation—these are not contextual background. They are the informational variables that specify the affordances skilled action is organised around. When they are removed, even partially, the player-environment system is no longer coupled to the affordance landscape of competition.

In handball, Bideau et al. (68) demonstrated this directly: handball goalkeepers' anticipatory responses deteriorated measurably when the natural kinematics of an approaching thrower were removed from a virtual reality task, even when the spatial geometry of the shooting situation was preserved—confirming that functional skill is coupled to action-specifying kinematic information, not surface resemblance to competition. In cricket, Pinder et al. (69) demonstrated that replacing a live bowler with a ball projection machine changed the informational source, and batters' movement initiation and coordination changed with it. The movement changed because the affordance changed.

The Foundations for Task Design (see Figure 2) model operationalises this. Its central node—*representative information*—is the standard against which every parameter adjustment is judged. A task is representative not when it looks like handball, but when the perceptual information it generates specifies affordances of competitive play: when the ball affords genuine shooting decisions; when opponents create real defensive pressure; when direction, space, and time produce the demands of the game; and when teammates and decision-making configurations create the relational complexity handball requires. Any modification that undermines this criterion, however tactically clever, trades short-term training effects for a loss of representative fidelity.

A modified 1v1 in the side sector illustrates representative design at its simplest viable scale. A backcourt player receives a pass from a feeder on his/her side and must adjust their pace, direction, and distance to a defender, who holds a ball in both hands, constraining their reach without eliminating their postural information, before deciding to shoot from distance, drive through, or feint. The goalkeeper responds to what emerges. The task contains no prescribed technique and no announced option: the informational substrate of the decision, e.g., the defender's foot placement, weight distribution, and lateral shift, is present and functional from the first repetition. The defender holding the ball is a precise parameter manipulation: it dampens the defender's reach affordances while preserving the postural information the attacker must learn to read, maintaining representative information-movement coupling without requiring full defensive freedom from early in the developmental arc^3^.

The same logic of representativeness governs the progression to 3v2 and 3v3 formats in the mid sector. In the 3v2, adjusting the players parameter to create numerical advantage ensures that opportunities for playing around, through, or over the defence arise frequently, making the affordance landscape richer and more legible for learners still developing their perceptual attunement. When the task becomes too easily solved, e.g., when the 3v2 advantage makes goals routine, introducing a delayed defender (e.g., performing a recovery run). This adds a representative time constraint on the 3v2 that the attackers can aim to exploit while allowing the two defenders to develop tactical delay strategies, not increasing the complexity of a drill but raising the representativeness of the constraint configuration to match the expanding capacities of the players. For more advanced players, however, the relevant challenge shifts entirely: numerical superiority is no longer given to be exploited but a condition to be created through movement, timing, and collective co-regulation.

Representativeness must also extend to the affective dimensions of performance. Headrick, Renshaw (70) have termed this affective learning design: the recognition that competitive handball carries an emotional and motivational dimension, e.g., the pressure of a close score, the consequence of a mistake in a decisive moment, that constitutes a further layer of environmental constraint shaping perception-action coupling. A player who has never had to read a defender's cues whilst managing the arousal of a close match in the final minutes has not fully developed the perception-action system that competitive handball demands. Designing learning environments that preserve the perceptual information of the game is therefore among the most consequential decisions a coach makes. Because skill is not in the player, nor in the drill, but in the functional relation between them, representative design is the practice of preserving that relation.

### Repetition without repetition

The principle of repetition without repetition carries a demanding methodological implication: it is insufficient to have players perform a skill repeatedly. What matters is how they repeat it. Tasks must be designed such that the learner is required to solve a movement problem rather than reproduce a movement template, and such that the problem is never quite the same twice. This means structuring practice so that variability is not incidental but functional: built into the task in ways that require the player to continuously explore the affordance landscape, generating novel coordinative solutions in response to shifting informational conditions rather than defaulting to a single established pattern.

Within the Foundations for Task Design model, repetition without repetition is achieved by maintaining invariance in the functional problem—the affordance—while systematically varying non-functional context ensuring that parameter combinations never fully stabilise. When the ball, opponent, decision-making, courtspace, and player configurations produce the same perceptual problem on every repetition, the learner is not practicing repetition without repetition, they are practicing repetition with repetition. Functional variability, variation that preserves the affordance while changing its embedding context, must be designed into the task structure itself, not left to chance.

A 1v1 in the wing sector makes this principle concrete. An attacking winger receives a pass from a feeder and approaches the wing position, but may use as many step-backs as needed before committing to the drive or jump-in. This is not an indulgence; it is a principled invitation to explore (71, 72). Each step-back resets the attacker's run-up, their relationship to the defender, and the informational configuration from which the movement solution must be composed. The defender's starting position is varied across repetitions, e.g., positioned higher up the court, lower, wider, so that the task problem is never identical. The defender's role here is revealing: not to prevent goals *per se*, but to create variability in the pressure experienced by the wing player. By varying starting position and press height, the defender becomes a source of perceptual information the attacker must learn to read, rather than an obstacle to be circumvented. What accumulates across these repetitions is not a fixed technique but a progressively refined capacity to attune to the defender's position and initiate the run-up when the available geometry affords penetration.

Rolling substitutions between attackers and defenders in exercises serves the same principle from the other direction. Because each player transitions continuously between roles, no two repetitions arrive in the same physical or attentional state. The task environment is in continuous low-level flux, which prevents the consolidation of a single fixed solution and maintains the exploratory character of the learning process throughout the session. The key conceptual move is to treat the practice task as a problem-space rather than a performance template: tasks presenting a clear functional goal, e.g., score, maintain possession, create a shooting opportunity against a recovering defence, while leaving the means to that goal genuinely open, allow the learner's movement system to explore, select, and progressively attune to the most functional solutions the environment affords (17).

### Manipulating constraints

Task constraint manipulation is where coaches make the most influential use of their pedagogical toolkit. Organismic constraints change slowly, and environmental constraints, particularly in indoor handball, are relatively fixed. Task constraints, by contrast, can be modified within and between sessions in ways that systematically reshape the affordance landscape players encounter and the movement solutions they explore.

The Foundations for Task Design model provides a structured framework for this. Its four parameters—ball, opponent(s), teammate(s), and decision-making—correspond directly to the task and environmental constraint categories in Newell's (23) model, and function as the practical levers through which coaches reshape the affordance landscape of practice. The model's central insight, and its most direct alignment with Newell's framework, is that these parameters are not independent levers but coupled dimensions of a single system. A modification to any one parameter propagates through the others, and the coach's task is to understand and anticipate these interactions rather than adjusting parameters in isolation.

**Figure 3:**
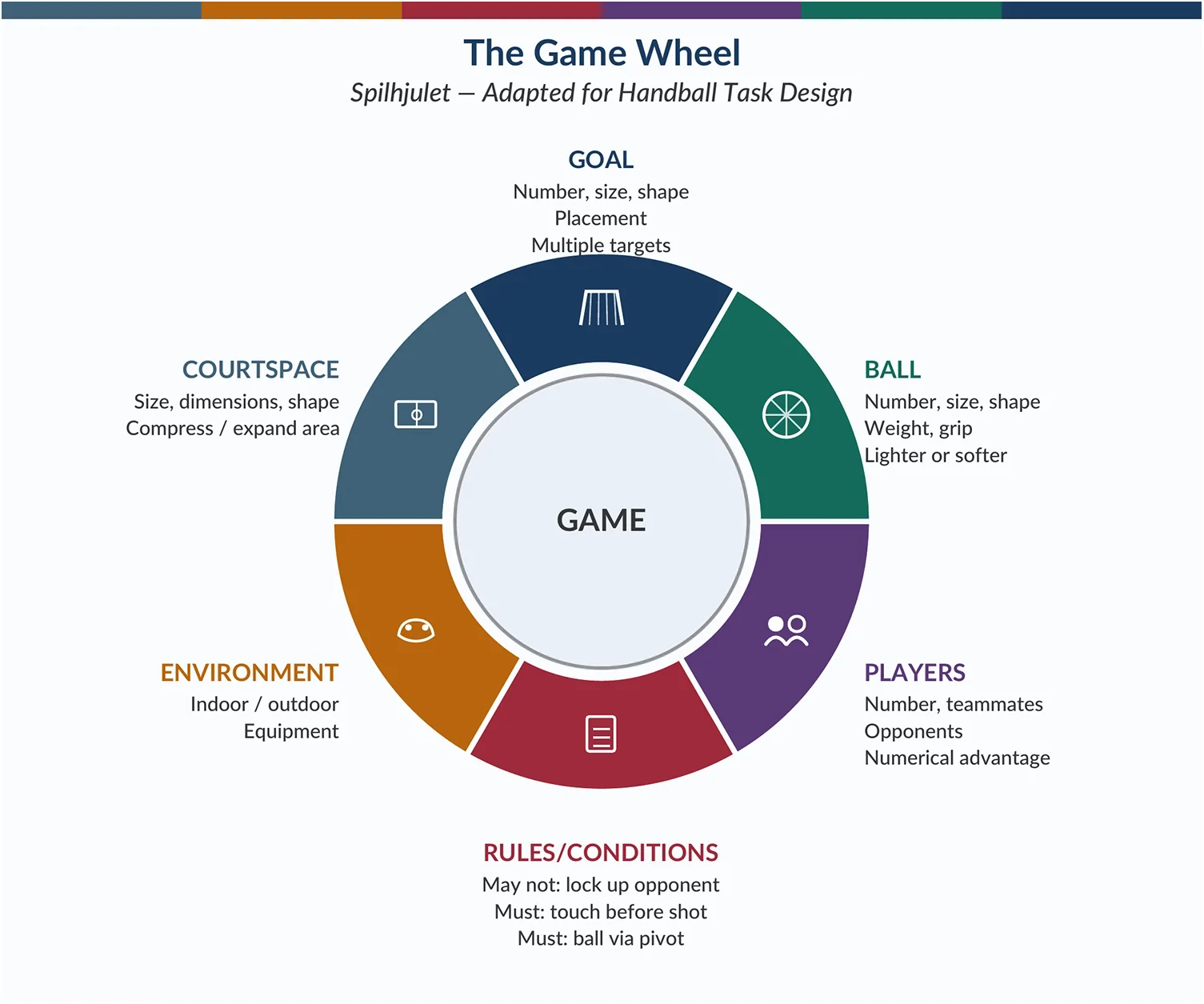
The Game Wheel [*Spilhjulet* (our translation); Eiberg & Siggaard (73)], adapted for handball task design. As a practical planning tool, the Game Wheel sits within the broader constraints-led approach to skill acquisition (1), operationalising Newell's task and environmental constraint categories into a coach-facing format. The model organises six planning parameters: goal; ball; players; rules/conditions; environment; and courtspace — around a central *game*. The wheel format makes visible the systemic coupling between parameters: a modification to any one spoke propagates through the others, reshaping the constraint configuration and affordance landscape that players encounter in practice.

A practical illustration of how this parameter logic has been translated into didactics is provided by the Danish model *Spilhjulet*—the Game Wheel [our translation] (73). Although originally designed for team ball games in physical education, its architecture maps directly onto the Foundations for Task Design model and translates without modification to handball as a structured planning and reflection tool for constraints-based practice design (see Figure 3).

Although the two models share an underlying parameter logic, they serve distinct functions: the Foundations for Task Design model (Figure 2) provides the theoretical criterion—representative information—against which every parameter adjustment is evaluated; the Game Wheel (Figure 3) provides the practical planning language through which coaches systematically modify and communicate constraint configurations in applied settings. Their compatibility demonstrates that this kind of parameter-based task thinking already has traction in coaching education, lending the Foundations model both practical precedent and pedagogical accessibility.

The Game Wheel sits within the broader constraints-led approach to skill acquisition (1). It organises six parameters: *goal*: the number, size, shape, and placement of scoring targets; *ball*: the number, size, shape, and weight of the ball in play; *players*: the number of participants and the numerical relationship between teams; *rules/conditions*: the behavioural stipulations governing the task; *environment*: the physical setting of the practice; and *courtspace*: the size, dimensions, and shape of the playing area—into a wheel whose spokes radiate from a central hub. The wheel format is deliberate: it makes visible the systemic coupling between parameters that a list format would obscure. When a coach adjusts one spoke, for example, compressing the pitch, the other spokes respond: ball-contact frequency rises, the number of meaningful opponent interactions per possession increases, shooting angles change, and the tactical problems confronting both attackers and defenders are transformed. The model equips coaches with a shared vocabulary for describing, discussing, and deliberately designing constraint configurations, and for reflecting on whether the changes they make move practice toward or away from the representative information criterion at the centre of the task design model.

Each parameter offers distinct affordance-shaping possibilities in handball. Modifying the conditions, e.g., disallowing locking-up (holding an opponent's body to neutralise them), requiring all players to touch the ball before a shot, or mandating that the ball must pass through the pivot, reshapes the task constraints without prescribing the specific movement solutions used to exploit them. By removing the affordance of physical immobilisation, defenders must open and close space through positioning and movement, inviting attackers through where they stand and how they shift, reorienting defensive skill development towards the perceptual and coordinative capacities that ecological dynamics identifies as constitutive of expertise. Varying goal parameters, such as using smaller goals to increase the precision demands on shooters and decision pressure on goalkeepers or introducing multiple targets to expand the spatial range of scoring options, directly modifies the informational landscape within which perception-action coupling is trained. Adjusting the ball, using a lighter, softer or smaller ball in developmental contexts to reduce the organismic constraints associated with limited grip strength, allows task demands to be scaled to the learner's current capacities. Modifying the pitch, e.g., compressing the playing area to intensify decision-making pressure, or extending it to create expansive attacking geometry, alters the spatial-temporal constraints within which movement solutions must be found. Adjusting the environment, e.g., moving a session outdoors, changing the playing surface, or varying the equipment available, changes the physical and perceptual conditions under which skills are practised. And manipulating the players parameter, for example, adjusting team numbers to create numerical advantages or disadvantages, pairing players of different positional specialisations, or introducing a floating player, changes the social and tactical affordance landscape within which collective coordination must emerge (56, 74).

To illustrate how the Game Wheel functions as a planning tool in handball practice, consider a coach working with youth players who are learning to exploit the six-metre line through the pivot. Beginning from a baseline 3v3 mid-sector format with a pivot, the coach identifies that the back-court players are not reading the pivot's angle-creation—they are passing to the pivot, or attacking in the space (s)he creates, only when the solution is too obvious, not when it is available. Using the Game Wheel, the coach adjusts the conditions parameter: a goal scored via a pivot touch is awarded double points, and no standing shots from beyond nine metres are permitted. The goal parameter remains unchanged (full-size goal, live goalkeeper). The ball parameter is unchanged. The pitch is slightly compressed in width to increase the frequency of pivot interactions. The players parameter remains 3v3 with pivot. The result is a task in which the pivot's positional affordances, for example, the angles they create, the defensive splits they produce, become more salient and more consequential for the attacker, without any prescription of when or how to use them. The perceptual-motor problem the task presents is more complex, more representative, and more precisely targeted than a general 3v3, yet it has been produced entirely through systematic parameter adjustment rather than through technical instruction.

Introducing the pivot or wing player into game situations illustrates how parameter manipulation shapes the emergence of shared affordances (62). In a 2v2 side-sector exercise with pivot, the back-court player's decision to drive, shoot, or feed is not independent of the pivot's movement: the pivot's angle-creation relative to the side-back player, their capacity to hold the defender down or exploit space, directly determines which affordances are available to the back-court player at any given moment. Neither player is executing a pre-planned role; each is continuously co-regulating their action in response to the same shared perceptual information, the defender's position, the developing geometry of the attack, and the quality of their collective play emerges from the precision of that co-regulation (32). Expanding to 4v3 and 4v4 formats extends this dyadic co-regulation to a three- and four-player collective system, where the pivot's positional relationship to the defence shapes not only the side-back player's options but the entire spatial-temporal organisation of the attack (75).

Coaches should approach this toolkit with reference to two complementary considerations. The first is the learner's intrinsic dynamics: the existing movement preferences, coordinative habits, and perceptual-motor tendencies the individual brings to the task (13). Effective constraint manipulation is calibrated to the player's current state, creating what Lev Vygotskij (1896–1934) (76), a contemporary and collaborator of Bernstein, termed a zone of proximal development: a task space at the productive boundary between what the learner can already accomplish reliably and what they are in the process of developing the capacity to accomplish (77), see also (78). Movement problems lying too far within the player's current competence will not invite exploration or provoke the reorganisation of perception-action couplings; those lying too far beyond it may produce disorganisation rather than adaptation (79). The coach's pedagogical skill lies precisely in this calibration.

The second consideration is representativeness. Constraint manipulation is not an end in itself; it is a means of preparing players to perceive and act effectively within the affordance landscape of competitive handball. Every constraint modification must be evaluated not only for its proximal effect on the player's current exploration but for its functional relationship to the perception-action demands of the game. Luteberget, Trollerud and Spencer (80) illustrate this tension clearly: both 3v3 and 6v6 game-based training conditions produced greater overall physical loading than mean match intensity, confirming their value as conditioning stimuli. Yet when the pitch size was held constant across conditions, the reduced player numbers in the 3v3 format forced wing and pivot players out of their position-specific roles, exposing them to movement demands and perceptual-motor problems different from those they encounter in competition. The physical overload had been achieved, but representativeness had been compromised. The principle of representative learning design and the principle of constraints manipulation are not in tension; they are mutually regulating. The question the coach must ask of every modification is not simply what does this train, but does what this trains belong to handball performance?.

## Timescale-sensitive task design: from mini-handball to full-scale competition

The three methodological principles above do not apply uniformly across all stages of development. The young child encountering handball for the first time inhabits a fundamentally different affordance landscape than the adolescent refining positional play in a youth elite programme, who in turn faces different developmental challenges than the senior professional expanding their perceptual-motor repertoire. An ecologically grounded account of skill development must therefore address not only the what of task design but the when: how the configuration of constraints appropriate to skill adaptation changes as the learner's intrinsic dynamics, perceptual capacities, and physical competencies develop across time (81).

At the earliest stages, the primary developmental task is not the acquisition of specific handball techniques but the establishment of the basic movement vocabulary and perceptual attunement from which handball-specific skills will eventually emerge. Task design should prioritise exploration and intrinsic motivation, with all task design parameters of the task design model scaled radically to the child's organismic constraints: smaller balls, lower goals, reduced playing areas, simplified rules, and small-sided formats that maximise ball contacts and interpersonal interactions. The representativeness criterion does not require that mini-handball resemble full-scale handball in its surface features; it requires that the perception-action couplings being developed, tracking a moving object, perceiving spatial relationships with teammates and opponents, timing a throwing action in relation to a defender's movement, are functionally relevant to the game the child will eventually play.

A developmentally sequenced programme begins with the simplest viable formats and progressively expands all task design parameters as player capacities consolidate. Children who cannot grip a regulation ball cannot explore throwing affordances; a pitch that is too large too soon reduces ball-contact frequency below the threshold needed for perceptual attunement to develop; and player numbers that exceed the child's current capacity for collective coordination produce tactical noise rather than tactical learning. In each case, the organism constraints of the learner are not being met by the task constraints of the environment—and the perception-action couplings that constitute handball skill cannot emerge under conditions that are systematically misaligned with the learner's current capacities (51).

Early sessions begin with 1v1 and progress through 3v2 and 3v3 formats to 3v3 with forward-return play. The key developmental task at this stage is learning to play around, through, or over the defence—the game's fundamental problem—in its most spatially and numerically legible form. Three outfield attackers against two or three defenders provides sufficient relational complexity to make the problem genuine, without the positional obligations and collective coordination demands of a full-team format that would exceed the learners' current capacities for collective attunement. As players move through adolescence and into structured competitive development, the affordance landscape expands. Organismic constraints shift substantially as athletic capacities grow: increased strength opens new shooting options, improved coordination enables more 1v1 duels, and the deepening perceptual repertoire allows players to attune to more subtle and temporally distal informational sources (24).

Introducing new playing positions, the pivot, for example, is not merely the addition of a player; it is the opening of a new affordance landscape. The pivot's presence at the six-metre line creates angles, defensive splits, and spatial options that do not exist in a three-back-player attack, and that require the back-court players to develop new perceptual attunements, to the pivot's angle relative to the defender, to the timing of the pivot's hold and release, to the haptic and visual signals indicating when the pivot has created usable space. Passing network analyses in handball confirm that the presence of a pivot reorganises the topology of collective coordination, shifting which players function as relational hubs, which connections are activated under defensive pressure, and where opportunities for penetration and combination are located (63).

Progressions may follow the same logic, introduce the new affordance landscape at a scale where it is sufficiently legible for the relevant perception-action couplings to develop, then expand player numbers as those couplings consolidate. Wing players are introduced subsequently, and with them the wing-entry affordance: the jump-in from the corner of the court, the narrow angle to goal, the 1v1 duel in the sector that requires a qualitatively different attunement to defender position and run-up timing than mid-sector or side-sector situations. Full small-sided play with and without pivot and forward-return play represents the culmination of the developmental arc: all positional roles present, full spatial complexity, continuous transitions between attack and defence, and the full consequence structure of competitive handball intact.

At the elite senior level, the task of skill development shifts from broad competency acquisition to the continuous expansion and refinement of the perceptual-motor repertoire. The elite player, in Bernstein's terms, can already solve the basic degrees of freedom problem in the most common situational configurations the game presents; what remains is the progressive attunement to less frequent, more subtle, or more demanding affordance structures, reading a defensive reorganisation at the periphery of attention, generating a functional shooting solution from an angle or distance outside the habitual repertoire, maintaining perceptual precision under the affective constraints of a high-stakes competition. Across all stages, each task design transition introduces new affordances and new perceptual-motor problems rather than simply making the existing problem harder. The coach's task across this arc and across the full developmental lifespan it prefigures, is to read where the player's current adaptive capacity lies and position the task at the boundary where new solutions are genuinely required, where repetition without repetition remains genuinely exploratory, and where the perceptual-motor system is still being asked to compose rather than retrieve.

## Reflections on tensions and limitations

The argument developed in this paper is presented with a degree of polemical clarity that serves its pedagogical aims but warrants some qualification. The contrast drawn between ecological dynamics and cognitivist frameworks is, in part, a rhetorical one: by articulating the distinctive commitments of an ecological account with precision, it becomes possible to identify what cognitivist approaches struggle to explain and what an ecological reformulation makes visible. This should not be read, however, as a claim that cognitivist research has yielded nothing of value. Decades of work in motor learning (e.g., on practice scheduling, feedback, retention, and the conditions supporting transfer) have produced empirically robust findings that practising coaches and sport scientists draw on with good reason (7, 8, 10). The theoretical dispute concerns the most appropriate conceptual framing for the phenomena these traditions study, not whether the phenomena themselves are real or the findings spurious. Bruineberg, Withagen and Van Dijk (37) have argued persuasively for productive pluralism within ecological psychology itself, an openness to multiple and sometimes competing framings as a mark of the field's maturity rather than its fragmentation. A similar spirit of pluralism applies here: an ecological dynamics perspective and a cognitivist one may, in many cases, generate overlapping practical recommendations, and the disagreement lies less in what coaches should do than in why they should do it. It is also worth acknowledging that the theoretical landscape extends beyond this particular binary. Barker, Nyberg and Larsson (44), working from a kinesio-cultural perspective, have raised important questions about whether even ecological dynamics frameworks fully account for the dispositional, tacit, and culturally embedded dimensions of skilled performance, a challenge from within the broader family of non-cognitivist accounts that points to the ongoing richness of theoretical debate in this area.

A second limitation concerns the operationalisation of the framework's central concepts. Affordances are, by definition, relational and emergent: they cannot be specified in advance as a fixed list of perceptual cues, nor reduced to observable environmental features independent of the organism encountering them. This theoretical richness is precisely what makes the concept powerful, but it also makes it difficult to operationalise for coaching in any straightforward way. The Foundations for Task Design and Game Wheel models, introduced here as a pedagogical bridge between principle and practice, necessarily simplifies: they provide a tractable planning and reflection language, but coaches who engage with the models should understand that they are heuristics, not formulas. Designing genuinely representative tasks requires sustained engagement with the framework's underlying commitments, not simply working through a checklist. Recent work has proposed alternative heuristic frameworks for scaling activity realism in coaching, including multi-dimensional accounts of fidelity across affective, conceptual, action, and physiological dimensions (82), which further illustrates the gap between the theoretical elegance of representativeness as a concept and its practical operationalisation for coaches. As O'Sullivan et al. (65) have noted, improving the communication of key ecological concepts to practitioners remains an ongoing challenge, and this paper should be understood as contributing to rather than completing that conversation.

Third, and relatedly, the applied and developmental sections of this paper occupy a position on the spectrum between theoretical implication and empirically validated recommendation that is not always explicit. This is a conceptual paper, and the developmental sequence proposed in the “Timescale-sensitive task design” section is grounded in the theoretical principles of ecological dynamics and in the authors' extended practical experience in handball coaching and education, rather than in a body of handball-specific longitudinal research. Where individual claims are supported by handball empirical work, for example, on kinematic variability in the jump shot and different throwing techniques, on physical loading in game-based training formats, or on goalkeeper anticipation, citations are provided. Where they are not, the recommendations should be understood as principled extrapolations from theoretical premises and broader sport science research, not as established findings from the handball context. The distinction matters: principled extrapolations are legitimate and necessary in applied theoretical work, but they carry a different epistemic weight, and future empirical work could test the developmental and task design claims advanced here in handball-specific populations and settings.

Finally, the framework makes significant demands on practitioners. Reconceptualising skill as a relational, emergent property of the performer-environment system, rather than a stored programme to be refined through repetition, requires coaches to relinquish familiar categories and familiar justifications for familiar practices. The theory-to-practice divide in sport coaching is well-documented (7), and the persistence of reductionistic approaches in coaching education reflects not only theoretical conviction but entrenched habits, institutional expectations, and the intuitive appeal of methods that appear to work in training even when they may not transfer to competition (83). An ecological dynamics account offers a more coherent theoretical language for how skilled handball performance can be conceptualised, but the practical and cultural conditions that would support its uptake across coaching education systems remain underdeveloped. This paper is addressed as much to that educational project as to the academic community and should be read in the context of an ongoing effort to develop theoretical frameworks that are both intellectually rigorous and practically accessible to those who coach the game.

## Concluding thoughts

This paper has proposed an ecological dynamics account of skill adaptation in handball that departs from the sport science frameworks historically dominating coaching education and sport science. Against the picture of the player as a composite of separable physical, technical, and tactical components, we have articulated an account in which skill is the continuous, context-sensitive self-organisation of perception and action in response to the interacting constraints of the game. Drawing on Bernstein, Newell, and Gibson, we have developed a conceptualisation of skill adaptation operating simultaneously at the level of the individual player, the attacker-defender dyad, and the collective team system, exemplifying principles across the developmental arc from children's first encounters with the sport to elite performers refining their craft.

Skill in handball, on this account, is not a fixed property of the player but a relational one, realised in the performer-environment system through the coupling of the player's perceptual-motor capacities and the affordance structure of the performance context. An expert is not someone who has acquired capacities in isolation but someone who has become more extensively and flexibly attuned to the informational landscape of the game: who can perceive more affordances, perceive them earlier and more precisely, and compose functional movement solutions from a wider range of coordinative configurations. Dexterity, not technical correctness, is the hallmark of handball expertise.

For practice, the implications are equally significant. A coach who understands that skill is constituted by perception-action coupling will design training differently from one who understands it as the storage and retrieval of motor programmes or as the sum of adaptations produced by isolated training modalities. They will ask, of every session and every task: what perceptual information does this preserve, and what does it remove? What affordances does this configuration invite the player to perceive? What movement solutions does this constraint landscape make available, and are they the solutions the game demands? They will understand that variability in movement is not noise to be eliminated but the adaptive medium through which functional skill is expressed, and they will design tasks that invite exploration and reward functional solutions rather than penalising deviations from a prescribed template.

Performing is adapting. The player who executes effectively in handball does not reproduce a fixed technique but composes a functional movement solution from the informational resources the situation provides, continuously and in real time. If handball is to develop coaches and players genuinely capable of meeting the demands of the game at its highest levels, it needs a theoretical foundation adequate to the complexity of what those demands are. We have argued that ecological dynamics provides precisely that foundation, not as a replacement for the practical wisdom experienced coaches have accumulated across careers of attentive engagement with the game, but as a theoretical language capable of making that wisdom explicit, communicable, and generative. So, too, is coaching.

## Footnotes

^1^It is important to clarify what this paper does and does not argue. We do not seek to dismiss cognitivist accounts of motor learning and control, nor to deny the role of cognition in skilled movement. Cognitive processes – attention, anticipation, decision-making, emotional regulation – can be considered consequential dimensions of handball performance. Our argument, rather, is that cognition is best understood as one of many interacting constraints shaping movement and action, not as a central executive that retrieves and runs pre-stored motor programmes (9). The separation of cognition, perception, and action into discrete, independently operating components is, from an ecological dynamics perspective, an analytical construction – a cutting apart of processes that are, in the living, moving player, inherently entangled and mutually constitutive. We argue, that an ecological dynamics conceptualisation has far-reaching practical consequences for how coaches and educators design sessions, tasks, and learning environments – consequences that cognitivist frameworks, however theoretically legitimate on their own terms, are less well equipped to generate.

^2^Ingold goes beyond Gibson’s relatively stable environment that affords to show how the environment itself is in flux, a meshwork of materials and forces. The skilled practitioner joins this flow rather than reading it. Ingold’s practitioner is constituted by movement itself. Identity and knowledge are produced in motion, not prior to it. If Gibson asks what the environment offers, Ingold asks what it means to move through a world you are always already part of.

^3^Removing the tackle is also a constraint manipulation that amplifies the demands on the defender’s positional play, postural control, and movement quality as the means of creating pressure and opening or closing defensive gaps.
